# Deep learning assisted retinal microvasculature assessment and cerebral small vessel disease in Fabry disease

**DOI:** 10.1186/s13023-025-03627-1

**Published:** 2025-04-03

**Authors:** Yingsi Li, Xuecong Zhou, Junmeng Li, Yawen Zhao, Yujing Yuan, Bo Yang, Jingjing Xu, Qijie Wei, Xiaoming Yan, Wei Zhang, Yuan Wu

**Affiliations:** 1 Department of Ophthalmology, Peking University First Hospital, Peking University, Beijing, 100034 China; 2 Department of Neurology, Peking University First Hospital, Peking University, Beijing, 100034 China; 3 Internet Institute, Beijing Normal University, Beijing, 100875 China; 4 Visionary Intelligence Ltd., Beijing, 100080 China

**Keywords:** Fabry disease, Microangiopathy, Retinal microvascular parameters, Cerebral small vessel disease, Brain lesions

## Abstract

**Purpose:**

The aim of this study was to assess retinal microvascular parameters (RMPs) in Fabry disease (FD) using deep learning, and analyze the correlation with brain lesions related to cerebral small vessel disease (CSVD).

**Methods:**

In this retrospective case control study, fundus images from 27 FD patients and 27 age- and sex-matched healthy subjects were collected. RMPs, encompassing diameter, density, symmetry, bifurcation, and tortuosity, were quantified. Laboratory examination results, Mainz severity score index (MSSI) scores, and a brain magnetic resonance imaging scan for CSVD scores were extracted and their relationships with RMPs was analyzed.

**Results:**

Utilizing artificial intelligence-assisted analysis, compared with controls, FD patients exhibited reduced diameter (*p* = 0.001 for central retinal artery equivalent, *p* = 0.049 for central retinal vein equivalent), density (*p* < 0.001 for vessel area density, *p* = 0.001 for length density), fractal dimension (*p* < 0.001), and heightened arteriolar and venular asymmetry ratios (*p* = 0.002 and *p* = 0.037, respectively), venular curvature tortuosity (*p* = 0.037), and simple tortuosity (*p* = 0.037) in retinal microvascular networks. Gender-based differences in RMPs were observed among FD patients. Furthermore, RMPs were significantly associated with disease markers such as plasma globotriaosylsphingosine and α-galactosidase A activity, as well as MSSI scores. Notably, there was a significant negative correlation between the arteriolar asymmetry ratio and CSVD-related scores (age-related white matter changes: *r* =  − 0.683, *p* = 0.001; Fazekas: *r* =  − 0.673, *p* = 0.001; Lacuna: *r* =  − 0.453, *p* = 0.045; small vessel diseases: *r* =  − 0.721, *p* = 0.012; global cortical atrophy: *r* =  − 0.582, *p* = 0.009).

**Conclusions:**

Fabry disease patients demonstrated increased vascular tortuosity and asymmetry, reduced density and diameter, and a simpler fractal dimension in retinal microvasculature. These microvascular characteristics may serve as preliminary indicators for assessing brain lesions and could represent potential novel biomarkers for CSVD, aiding in the monitoring of FD severity and progression.

**Supplementary Information:**

The online version contains supplementary material available at 10.1186/s13023-025-03627-1.

## Introduction

Fabry disease (FD, OMIM #301500) is a rare, potentially lethal X-linked disorder, with an incidence of 1 in 40,000 to 1 in 117,000 live births [1, 2]. This condition arises from a genetic deficiency in the lysosomal enzyme α-galactosidase A (α-Gal A) due to mutations in the GLA gene [1]. The consequent reduction in α-Gal A enzyme levels and functionality leads to the progressive accumulation of lysosomal globotriaosylceramide (Gb3) in various cells and tissues, culminating in irreversible cellular dysfunction [1, 3]. This accumulation triggers tissue inflammation and fibrosis, ultimately leading to multi-organ abnormalities, including progressive kidney failure, arrhythmias, and cardiomyopathy, etc. [1].

The accumulation of Gb3 leading to endothelial dysfunction, dysregulation of the nitric oxide pathway, or microvascular remodeling can all directly affect the function of microvessels[4].

It also diminishes blood capacity to withstand hydrostatic pressure, precipitating the formation of aberrant vascular networks that could impair tissue perfusion and precipitate cellular malfunction [5–8]. In ocular system, retinal and conjunctival vascular abnormalities are prevalent and highly significant in FD [9–12]. Notably, enhanced vessel tortuosity in fundus images from FD patients has been documented, correlating intimately with systemic disease progression and severity markers, such as the Mainz Severity Score Index (MSSI) [13–15]. Despite this, research on retinal vascular networks in FD, especially concerning vascular extension, remains sparse [13–17]. The advent of advanced artificial intelligence (AI) in medical imaging has marked a transformative epoch. Our team has previously engineered a deep learning (DL) model that automates the segmentation of retinal vasculature and quantifies retinal microvascular parameters (RMPs), leveraging the Inception-V3 architecture and Atrous Spatial Pyramid Pooling module [18]. In our current study, we have conducted a thorough and expeditious evaluation of RMPs, encompassing retinal microvascular morphology, branch geometry, and network extension characteristics, utilizing fundus photographs to track the severity and progression of FD patients.

Microvascular lesions usually involve the brain in Fabry, with cerebral small vessel disease (CSVD) being the primary manifestation of cerebral involvement in FD patients [19]. Our previous cohort study indicated that the incidence of CSVD in FD was as high as 62% [20]. The main manifestations of CSVD on brain magnetic resonance imaging (MRI) include white matter hyperintensities (WMHs), cerebral microbleeds (CMBs), lacunar infarcts (LIs), and enlarged perivascular spaces (EPVSs). Nervous system involvement is one of major causes of reduced life expectancy and high mortality in FD. Recently, the relationship between retinal microvascular network and CSVD has emerged as a new focus in medical research. Increasing evidence supported retinal microvascular changes as potential biomarkers of CSVD, due to the similarity between retinal and brain microvasculature in terms of embryonic origin, anatomy, and physiological function such as blood–retina barrier and glial cell connections [21, 22]. For instance, in patients with diabetes, studies have shown that decreased retinal vessel density was significantly associated with cognitive decline, stroke risk, and CSVD markers on MRI [23, 24]. Although the relationship between retinal microvascular network and CSVD lesions has been extensively studied and confirmed in various diseases such as Alzheimer’s disease and diabetes [23–25], the specific relationship between the imaging features of CSVD and RMPs in FD patients has not been discussed.

Consequently, the purposes of this study were twofold: (1) to analyze the fundus vascular characteristics of Fabry disease with DL algorithms, and (2) to evaluate the correlation between RMPs and FD biomarkers and MRI lesions of CSVD to provide new insights into disease prognosis.

## Methods

### Study population

This investigation was a retrospective case–control study conducted at Peking University First Hospital. Adherence to the principles outlined in the Declaration of Helsinki, and our protocol was approved by the local institution’s Review Board. Twenty-seven patients diagnosed with FD at Peking University First Hospital between 2016 and 2021 were included in FD group. The diagnosis was based on a comprehensive evaluation, incorporating the presence of characteristic clinical manifestations, a positive family history, and important laboratory test results. Data on demographics, types of GLA mutations, plasma laboratory results such as α-Gal A and globotriaosylsphingosine (Lyso-Gb3), medical history, treatment procedures, and disease severity evaluation such as Mainz Severity Score Index (MSSI) were collected. The control group was aged and gender-matched healthy subjects with ocular refraction <  − 3.00 D, who participated in routine physical examinations at Peking University First Hospital. The age difference was less than 2 years old. Those with systemic conditions such as diabetes and primary hypertension, as well as ocular diseases such as severe myopia, glaucoma, and cataracts, were excluded. Informed consent for all examinations was obtained from all included participants.

### Ophthalmological examination

**Fig. 1:**
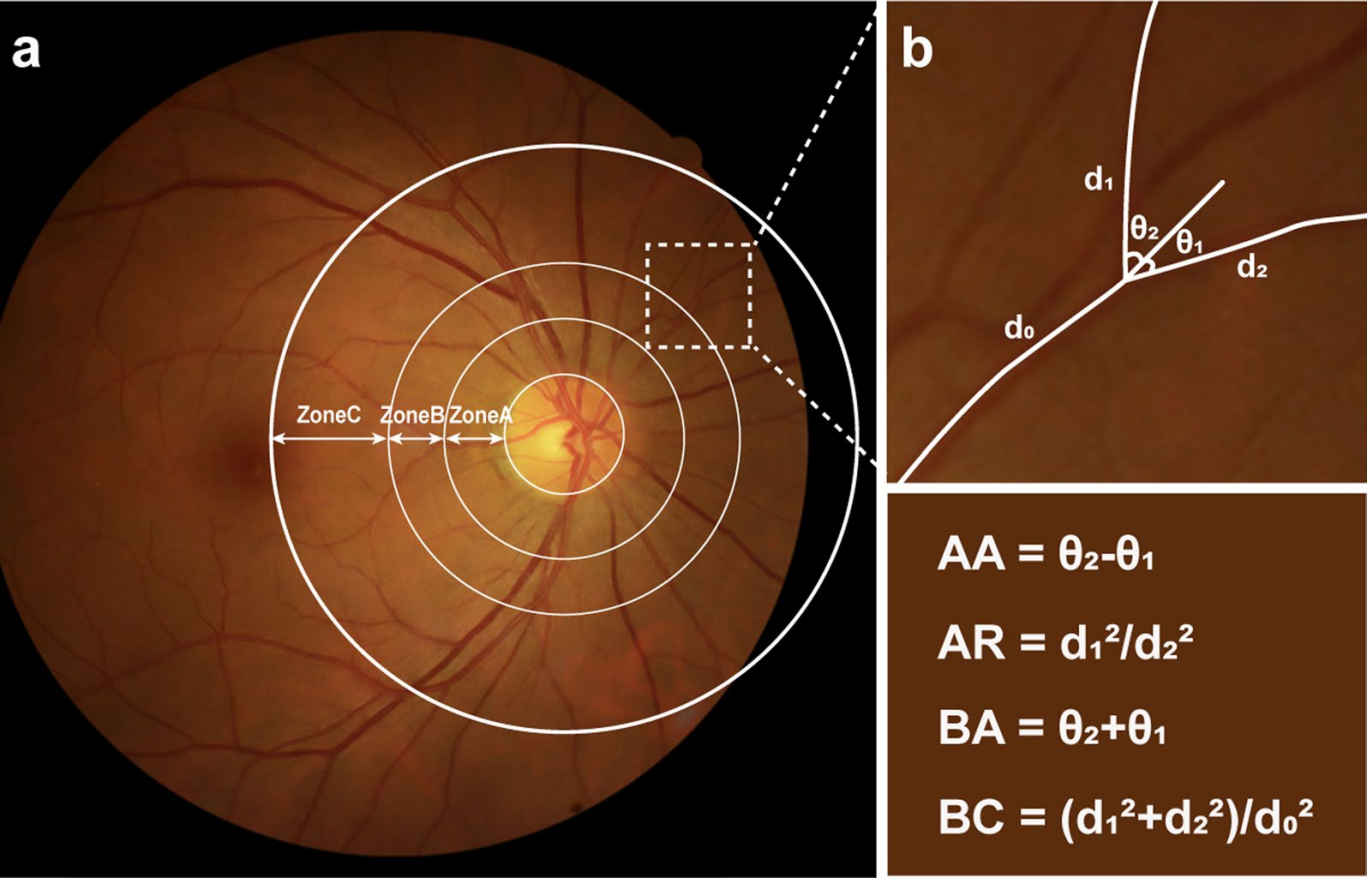
Fundus image partitions and vessel bifurcations. **a** Image partitions from one healthy subject with three zones. Zones A, B, and C were the annular area with an extension of 0.5, 0.5, and 1-disc diameter, respectively. **b** The above vessel at the bifurcation in Zone C and the following parameter calculation formulas. AA: angle asymmetry; AR: asymmetry ratio; BA: Branching angle; BC: Branching coefficient; d0: diameter of the trunk vessel; d1 and d2 (d1 > d2): diameter of two daughter (branching); θ1 and θ2 (θ1 < θ2): branching angle

The routine ophthalmological examinations of all subjects at their first visit to the ophthalmology clinic were retrospectively reviewed. Intraocular pressure, visual acuity assessment, and fundus photography were performed by a trained technician, while anterior segment slit-lamp examination was conducted by the same ophthalmologist. Retinal photographs centered at the posterior pole using a 45° digital fundus camera (CR2AF, Canon Inc., Kanagawa, Japan) of 54 participants were collected and reviewed. Before obtaining quantitative parameters, the fundus image was divided into three zones (Zone A, Zone B, and Zone C) according to the Singapore I Vessel Assessment, as shown in Fig. 1a [26]. In this research, we harnessed a trained DL model for automatic retinal vascular segmentation and RMPs analysis system with U-Net architecture (Visionary Intelligence Ltd., Beijing, China) [18]. The model featured the Inception-V3 as its encoder and integrated the Atrous Spatial Pyramid Pooling module to enhance feature extraction [18]. This system enabled precise differentiation between arteries and veins, facilitated by the softmax function, thereby facilitating the quantification of RMPs.

### Retinal vascular morphology assessment

All RMPs (Table 1) in different zones were calculated by specific formulas or schemes. Firstly, for observation of the variability of vascular diameter, the standard deviation (SD) of the six largest arterioles and venules in Zone B were measured [27]. The central retinal artery and vein equivalent (CRAE and CRVE) was computed using the enhanced Knudston-Parr-Hubbard equation, representing the equivalent single-vessel parent width for the six largest arterioles and venules, followed by the calculation of the arteriole-to-venule ratio (AVR) based on their proportion [28]. According to previous studies, vessel length density (VLD) was determined by calculating the ratio of vessel length to the area of the skeletonized image, and vessel area density (VAD) was quantified as the percentage of the total measured area occupied by blood flow within the vessel area [29, 30]. The ratio of the vessel’s length at midpoints of two bifurcations to the parent vessel’s diameter at the first bifurcation was denoted as the length-diameter ratio (LDR) [31].

**Table 1:** Description of all RMPs measured in different zones

Parameter	Description	Abbre	Zone	Ref
Diameter	Standard deviation of arteriole /venule	SDa/ SDv	B	[27]
	Central retinal artery equivalent	CRAE	B	[28]
	Central retinal vein equivalent	CRVE	B	[28]
Density	Vessel length density	VLD	C	[29]
	Vessel area density	VAD	C	[30]
Ratio	Length-diameter ratio	LDRa/ LDRv	C	[31]
	Arteriovenous ratio	AVR	B	[28]
Asymmetry	Optimality deviation	ODa/ ODv	C	[26]
	Angle asymmetry	AAa/ AAv	C	[32]
	Asymmetry ratio	ARa/ ARv	C	[32]
Bifurcation	Branching angle	BAa/ BAv	C	[32]
	Branching coefficient	BCa/ BCv	C	[32]
	Junctional exponent deviation	JEDa/ JEDv	C	[26]
	The number of first branching	NFBa/ NFBv	C	[27]
Tortuosity	Curvature tortuosity	CTa/ CTv	C	[26]
	Simple tortuosity	STa/ STv	C	[26]
	Fractal dimension	FrD	C	[26]

RMPs, retinal microvascular parameters; The parameter names ending in lowercase ‘a’ or ‘v’ correspond to the arteriolar network or venular network, respectively.

### Retinal vascular extension assessment

One typical retinal vessel at a bifurcation was first selected to obtain the asymmetry and bifurcation parameters, including angular asymmetry (AA), asymmetry ratio (AR), branching angle (BA), and branching coefficient (BC). The calculations (Fig. 1b) were described in a previous study conducted by Sun et al. in detail [32]. The number of first branches of retinal vessels (NFB) denoted the quantity of arterioles and venules that underwent a primary bifurcation or a daughter vessel within Zone C [27], and the calculation of junctional exponent deviation (JED) was based on the average count of initial branching vessels [26]. Optimality deviation (OD) was a new metric to assess the degree of deviation from ideal conditions to help in evaluating the optimality of the connection between parent and daughter vessels in a bifurcation, introduced by Witt NW and colleagues [26]. As stated previously, measurements of curvature tortuosity (CT) and simple tortuosity (ST) indicated the tortuosity of arterioles and venules, and the fractal dimension (FrD) signified the intricate geometric formations of the retinal vascular fractal [26].

### Brain MRI scan and CSVD assessment

For this retrospective study, brain MRI data on 3 T *GE* 1.5 Signa *Twin Speed* scanners (GE Healthcare, Waukesha, USA) from FD patients were extracted. Then, the rating scales of CSVD lesions and brain atrophy were assessed by two independent experienced physicians. When there was conflicting data, the two physicians negotiated and reached an agreement. WMHs were evaluated by Fazekas with a scale from 0 to 3 [33] and age-related white matter change (ARWMC) scores [34], and CMB was measured by a reliable microbleed anatomical rating scale (MARS) [35]. The EPVS scores in the basal ganglia and centrum semiovale were calculated separately based on the coded approach outlined by Doubal FN et al. [36]. The comprehensive small-vessel disease score (SVDS) (0–4) and global cortical atrophy (GCA) scale for brain atrophy were also assessed [37, 38].

### Statistical analyses

The normality of the distribution for all continuous variables were assessed using the Shapiro–Wilk test. An unpaired Student’s t test was conducted to compare RMPs between FD and control groups and males and females when the data (mean ± SD) followed a normal distribution. The Mann–Whitney U test was applied and presented as the median (25th percentile, 75th percentile). Receiver operating characteristic (ROC) curve analysis was performed to assess the ability of RMPs to discriminate between mild (MSSI score < 20) and moderate-to-severe (MSSI score ≥ 20) FD patients. The correlation of RMPs with disease markers, severity, and CSVD-related indices was performed by Spearman’s rank-order method. To mitigate bias, the analysis was conducted only on the right eye of all participants, including both FD patients and control groups. Statistical analyses were carried out using SPSS version 26.0 for Windows (IBM, Chicago, USA) with significance set at two-sided *p* < *0.05*.

## Results

### Demographics and clinical features of patients with FD

Table 2 shows the demographic and clinical features of the FD patients in the FD group. A total of 27 patients were included, with 19 being male and 8 females, originating from 14 separate families, and their ages spanned from 16 to 73 years. Their onset age ranged from 5 to 18 years old. Among them, 6 patients had hypertension, and only two patients had vision impairment. The mean concentrations of α-Gal A activity (of 22 patients) and Lyso-Gb3 (of 15 patients) in FD patient serum were 4.04 ± 7.27 μmol/L/h and 59.66 ± 54.26 ng/ml, respectively. The overall MSSI scores and MRI data of 5 patients were unavailable. The MSSI scores ranged from 5 to 42, and the age of patients who underwent MRI examination ranged from 7 to 62 years. The median (with interquartile range) values for CVSD scores were as follows: 3 (2, 8) for ARWMC; 1 (0.75, 2) for Fazekas; 1 (0, 2) for EPVS; 0 (0, 2) for MARS; 0 (0, 1) for LI; 1 (0.5, 3) for SVDS; and 2 (0, 7.5) for GCA. In the present study, we also included 27 normal controls and there was no significant difference between FD and control groups in terms of age and sex (all *p* > 0.05).

**Table 2:** Demographics and clinical data of the FD patients

Patients	Family	Sex	Age	Onset age (years old)	α-Gal A	Lyso-Gb3 [ng/ml]	MSSI	Hypertension	Visual impairment
1	F1	M	23	7	0.8	97.8	13	Yes	Yes
2	F1	F	50	18	26	9.37	7	No	No
3	F2	M	16	9	1.8	76.93	6	No	No
4	F2	F	54	7	2.07	5.2	9	No	No
5	F3	M	46	7	0.9	51.71	34	No	No
6	F3	F	49	7	5.88	2.99	18	Yes	No
7	F4	F	73	5	21.4	NA	28	No	No
8	F4	M	41	9	0.6	200	33	No	No
9	F5	M	23	9	0.24	81.23	19	No	No
10	F6	M	34	10	0.31	32.47	19	No	No
11	F6	F	40	7	2.54	3.08	11	No	No
12	F6	F	64	10	2.37	3.31	18	Yes	No
13	F7	M	35	7	0.1	NA	36	No	No
14	F7	M	16	6	1	NA	10	No	No
15	F8	F	37	5	16.4	NA	5	No	No
16	F8	M	60	8	0.8	NA	42	Yes	No
17	F9	M	30	9	0.7	55.34	23	Yes	Yes
18	F9	M	46	7	0.61	105.12	10	No	No
19	F10	M	35	10	0.3	89.18	20	No	No
20	F11	M	36	5	2.4	NA	33	No	No
21	F12	M	21	5	1.4	NA	20	No	No
22	F13	M	39	7	0.23	81.14	38	Yes	No
23	F14	M	53	NA	NA	NA	NA	NA	NA
24	F14	F	28	NA	NA	NA	NA	NA	NA
25	F3	M	17	NA	NA	NA	NA	NA	NA
26	F5	M	51	NA	NA	NA	NA	NA	NA
27	F6	M	32	NA	NA	NA	NA	NA	NA

Patients	MRI examination age	ARWMC	Fazekas	EPVS	MARS	Lacuna	SVDS	GCA
1	16	2	1	1	0	0	1	2
2	43	14	3	2	NA	0	NA	6
3	16	0	0	0	0	0	0	0
4	57	0	0	0	NA	0	NA	0
5	36	6	2	2	0	1	1	5
6	39	6	2	0	NA	1	NA	6
7	62	14	3	1	NA	0	NA	13
8	34	4	1	2	0	0	1	4
9	21	8	2	3	0	0	2	14
10	25	2	0	0	NA	0	NA	0
11	30	2	1	0	NA	0	NA	0
12	57	18	3	2	NA	3	NA	15
13	26	0	0	1	0	0	0	0
14	7	0	0	1	0	0	0	0
15	29	2	1	1	NA	0	NA	0
16	50	14	3	2	4	3	3	7
17	24	4	1	0	0	0	1	2
18	39	8	2	2	2	4	3	11
19	27	4	1	2	4	2	3	0
20	26	2	1	1	NA	0	NA	0
21	15	2	1	1	0	0	1	NA
22	37	2	1	3	2	1	3	8
23	NA	NA	NA	NA	NA	NA	NA	NA
24	NA	NA	NA	NA	NA	NA	NA	NA
25	NA	NA	NA	NA	NA	NA	NA	NA
26	NA	NA	NA	NA	NA	NA	NA	NA
27	NA	NA	NA	NA	NA	NA	NA	NA

FD, Fabry disease; F, female; M, male; α-Gal A, α-galactosidase A; Lyso-Gb3, globotriaosylsphingosine; MSSI, Mainz severity score index; MRI: magnetic resonance imaging; ARWMC, age related white matter changes; EPVS, enlarged perivascular spaces; MARS, microbleed anatomical rating scale; SVDS, small vessel diseases; GCA, global cortical atrophy; NA, data not available.

### Comparison of RMPs between the FD and control groups

As shown in Table 3, the retinal vessel density in the FD group was lower than that in the age-matched control group for both VAD (*p* < 0.001) and VLD (*p* < 0.001). Additionally, patients with FD exhibited significantly lower levels of CRAE (*p* = 0.001), CRVE (*p* = 0.049), and FrD (*p* < 0.001), while there were no significant differences between the two groups in terms of SD, LDR, and AVR. Upon examining the symmetry characteristic, we observed a notable decrease in AAv and an increase in ARa and ARv calculated scores, indicating a significant rise in vessel asymmetry among FD patients when compared to the control group (*p* = 0.003 for AAv; *p* = 0.002 for ARa; *p* = 0.037 for ARv). The FD group also exhibited a decreased BAa, although this disparity did not reach statistical significance in the remaining bifurcation indices. FD patients had a higher retinal venular tortuosity (*p* = 0.037 for CTv and STv), with a fold change of 1.25 of venular curvature tortuosity. While no differences were observed in the arterioles.

**Table 3:** The values of RMPs in two groups of the right eye

Parameters		FD group (*n* = 27)	Control group(*n* = 27)	*P* value
*Retinal vascular morphology*				
Diameter	SDa^‡^	15.39 (10.56, 18.74)	13.15 (10.27, 19.31)	0.723
	SDv^†^	25.02 ± 7.97	25.08 ± 7.09	0.977
	CRAE^†^	138.45 ± 26.16	159 ± 18.15	0.001^**^
	CRVE^†^	226.41 ± 29.14	240.51 ± 21.76	0.049^*^
Density	VLD^†^	0.03 ± 0.01	0.04 ± 0.005	< 0.001^***^
	VAD^†^	0.11 ± 0.02	0.13 ± 0.01	< 0.001^***^
Ratio	AVR^‡^	0.63 (0.56, 0.70)	0.64 (0.62, 0.70)	0.272
	LDRa^‡^	12.71 (7.45, 23.11)	8.81 (4.90, 14.71)	0.172
	LDRv^‡^	8.27 (4.17, 9.57)	10.37 (7.13, 13.51)	0.167
*Retinal vascular extension*				
Asymmetry	AAa^†^	34.73 ± 13.37	32.14 ± 11.14	0.460
	AAv^‡^	26.01 (19.16, 36.73)	38.96 (31.14, 46.42)	0.003^**^
	ARa^†^	0.59 ± 0.17	0.45 ± 0.13	0.002^**^
	ARv^‡^	0.41 (0.28, 0.53)	0.32 (0.27, 0.36)	0.037^*^
	ODa^‡^	0.14 (0.09, 0.20)	0.14 (0.09, 0.21)	0.816
	ODv^‡^	0.09 (0.06, 0.13)	0.12 (0.09, 0.17)	0.098
Bifurcation	BAa^‡^	77.51 (68.94, 91.02)	87.30 (77.23, 105.04)	0.041^*^
	BAv^‡^	85.26 (70.37, 114.93)	85.84 (74.24, 107.46)	0.883
	BCa^‡^	1.30 (1.01, 1.43)	1.32 (1.14, 1.49)	0.547
	BCv^‡^	1.04 (0.92, 1.15)	1.11 (0.92, 1.41)	0.295
	NFBa^‡^	3.00 (1.00, 4.00)	3.00 (3.00, 4.00)	0.052
	NFBv^‡^	3.00 (2.00, 4.00)	4.00 (3.00, 4.00)	0.421
	JEDa^‡^	− 0.07 (− 0.44, 0.46)	− 0.13 (− 0.35, 0.07)	0.509
	JEDv^‡^	0.31 (− 0.02, 0.57)	0.21 (− 0.17, 0.43)	0.197
Tortuosity	CTa^‡^	0.58 (0.42, 1.05)	0.46 (0.36, 0.52)	0.064
	CTv^‡^	0.71 (0.49, 1.19)	0.57 (0.51, 0.69)	0.037^*^
	STa^‡^	1.09 (1.08, 1.12)	1.08 (1.07, 1.10)	0.059
	STv^‡^	1.09 (1.09, 1.11)	1.08 (1.08, 1.10)	0.037^*^
	FrD^‡^	1.54 (1.50, 1.55)	1.58 (1.56, 1.58)	< 0.001^***^

RMPs, retinal microvascular parameters; FD, Fabry disease; SD, standard deviation; CRAE, central retinal artery equivalent; CRVE, central retinal vein equivalent; VLD, vessel length density; VAD, vessel area density; AVR, arteriovenous ratio; LDR, length-diameter ratio; AA, angle asymmetry; AR, asymmetry ratio; OD, optimality deviation; BA, branching angle; BC, branching coefficient; NFB, number of first branches; JED, junctional exponent deviation; CT, curvature tortuosity; ST, simple tortuosity; FD, fractal dimension. The parameter names ending in lowercase ‘a’ or ‘v’ correspond to the arteriolar network or venular network, respectively. ^†^ Data were displayed with Mean ± SD and analyzed by two-sided independent *t*-test; ^‡^ Data were presented as median (25th percentile, 75th percentile) and analyzed using a two-sided Mann–Whitney U test; ^*^p < 0.05, ^**^p < 0.01, ^***^p < 0.001.

### Gender subgroups analysis in systemic parameters and RMPs

**Fig. 2:**
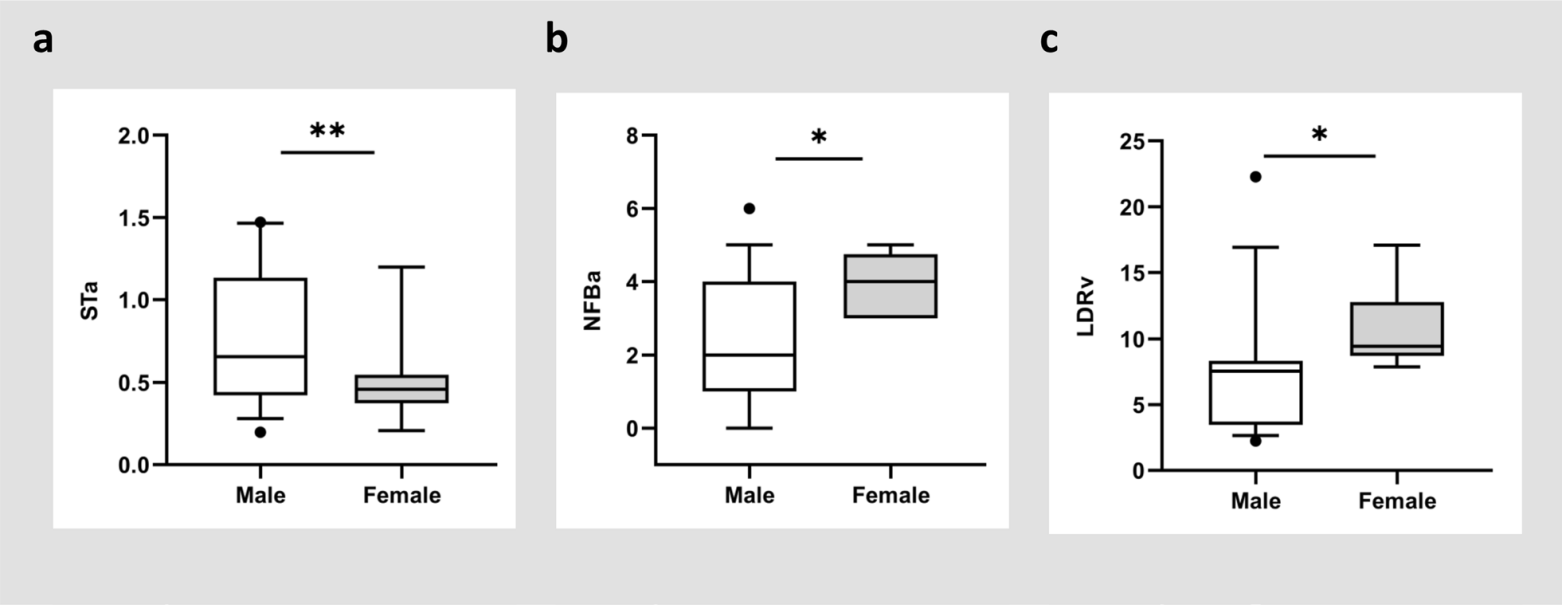
Gender subgroups analysis in RMPs of FD patients. Data were displayed by box plot (10th percentile to 90th percentile). The differences in STa (**a**) and NFBa (**b**) were analyzed by Student’s *t*-test and LDRv (**c**) was detected by Mann–Whitney. *p < 0.05, **p < 0.01

The clinical characteristics indicated that the age of 19 males (34.42 ± 13.04) was significantly younger than that of females (45.38 ± 14.60), with a p value of 0.014, while the onset age was not significantly different (Table 4). Consistent with our previous data, male patients had lower α-Gal A activity (0.70 [0.30, 1.00] vs. 5.88 [2.37, 21.40], *p* < 0.001) and higher Lyso-Gb3 activity (81.19 [54.43, 99.63] vs. 3.31 [3.04, 7.29], *p* = 0.002) than in female patients, but no effect of gender on brain MRI lesions was observed [20]. As shown in Fig. 2, the male FD cohort showed significantly higher retinal vessel tortuosity of STa, lower vascular branching of NFBa and LDRv (*p* = 0.001, *p* = 0.014, *p* = 0.032, respectively) in comparison to females (Table 5).

**Table 4:** Gender subgroups analysis in systemic parameters and MRI scores of FD patients

Parameters	Males (*n* = 19)	Females (*n* = 8)	*P*-value
Age^†^	34.42 ± 13.04	45.38 ± 14.60	0.014^*^
Onset-age (years old) ^‡^	7 (7, 9)	7 (5, 10)	0.716
α-Gal A^‡^	0.70 (0.30, 1.00)	5.88 (2.37, 21.40)	< 0.001^***^
Lyso-Gb3 [ng/ml] ^‡^	81.19(54.43, 99.63)	3.31 (3.04, 7.29)	0.002^**^
MSSI^†^	23.73 ± 11.48	13.71 ± 8.08	0.052
Hypertension^#^	4 (26.67%)	2 (28.57%)	0.311
Visual impairment^#^	2 (13.33%)	0	0.311
MRI examination age^†^	26.60 ± 11.14	45.29 ± 13.52	0.003^**^
ARWMC^‡^	2 (2, 6)	6 (2, 14)	0.264
Fazekas^‡^	1 (0, 2)	2 (1, 3)	0.130
EPVS^‡^	1 (1, 2)	1 (0, 2)	0.239
MARS	0 (0, 2)	NA	NA
Lacuna^‡^	0 (0, 1)	0 (0, 1)	0.798
SVDS	1 (0.5, 3)	NA	NA
GCA^‡^	2 (0, 7.25)	6 (0, 13)	0.586

MRI, magnetic resonance imaging; FD, Fabry disease; α-Gal A, α-galactosidase A; Lyso-Gb3, globotriaosylsphingosine; MSSI, Mainz severity score index; ARWMC, age related white matter changes; EPVS, enlarged perivascular spaces; MARS, microbleed anatomical rating scale; SVDS, small vessel diseases; GCA, global cortical atrophy; NA, data not available. ^†^ Data were displayed with Mean ± SD and analyzed by two-sided independent *t*-test; ^‡^ Data were presented as median (25th percentile, 75th percentile) and analyzed by two-sided Mann–Whitney U; # Data for categorical variables were presented as proportions and analyzed by Pearson chi-square test; ^*^p < 0.05, ^**^p < 0.01, ^***^p < 0.001

**Table 5:** Gender subgroups analysis in RMPs of FD patients

Parameters		Males (*n* = 19)	Females (*n* = 8)	*P*-value
*Retinal vascular morphology*				
Diameter	SDa^†^	16.53 ± 6.85	13.73 ± 2.96	0.279
	SDv^†^	23.93 ± 8.27	27.62 ± 6.99	0.280
	CRAE^‡^	137.21 (121.61, 143.57)	156.79 (126.11, 168.49)	0.100
	CRVE^†^	225.57 ± 27.38	228.41 ± 34.92	0.822
Ratio	AVR^†^	0.60 ± 0.14	0.67 ± 0.06	0.160
	LDRa^‡^	17.38 (10.34, 30.33)	9.31 (5.53, 21.45)	0.150
	LDRv^‡^	7.53 (3.47, 8.33)	9.42 (8.70, 12.76)	0.032*
Density	VLD^†^	0.03 ± 0.01	0.03 ± 0.004	0.295
	VAD^†^	0.10 ± 0.02	0.12 ± 0.02	0.204
*Retinal vascular extension*				
Asymmetry	AAa^†^	34.49 ± 14.04	35.21 ± 12.84	0.904
	AAv^‡^	24.96 (17.37, 36.73)	26.77 (24.26, 46.60)	0.243
	ARa^†^	0.60 ± 0.19	0.56 ± 0.14	0.606
	ARv^†^	0.43 ± 0.19	0.41 ± 0.14	0.747
	ODa^‡^	0.13 (0.07, 0.22)	0.14 (0.11, 0.16)	0.624
	ODv^‡^	0.09 (0.07, 0.14)	0.09 (0.05, 0.12)	0.426
Bifurcation	BAa^†^	78.35 ± 17.26	82.89 ± 18.59	0.560
	BAv^†^	91.12 ± 29.97	96.46 ± 34.05	0.688
	BCa^‡^	1.30 (1.05, 1.60)	1.29 (0.96, 1.34)	0.713
	BCv^‡^	1.11 (0.92, 1.17)	1.01 (0.85, 1.08)	0.339
	NFBa^†^	2.16 ± 1.74	3.88 ± 0.84	0.014^*^
	NFBv^‡^	3.00 (2.00, 4.00)	3.00 (2.00, 4.75)	0.782
	JEDa^†^	− 0.10 ± 0.59	0.13 ± 0.31	0.319
	JEDv^‡^	0.17 (− 0.04, 0.51)	0.41 (0.20, 0.61)	0.203
Tortuosity	CTa^‡^	0.66 (0.42, 1.13)	0.46 (0.37, 0.55)	0.149
	CTv^‡^	0.71 (0.56, 1.24)	0.64 (0.43, 0.87)	0.367
	STa^†^	1.11 ± 0.03	1.08 ± 0.01	0.001^**^
	STv^‡^	1.09 (1.09, 1.11)	1.09 (1.08, 1.11)	1.000
	FrD^†^	1.52 ± 0.04	1.54 ± 0.03	0.243

^†^ Data were displayed with Mean ± SD and analyzed by two-sided independent t-test;

^‡^ Data were presented as median (25th percentile, 75th percentile) and analyzed by two-sided Mann–Whitney U; ^*^p < 0.05, ^**^p < 0.01.

### Correlation analysis of RMPs with systemic parameters and CSVD-related scores

**Fig. 3:**
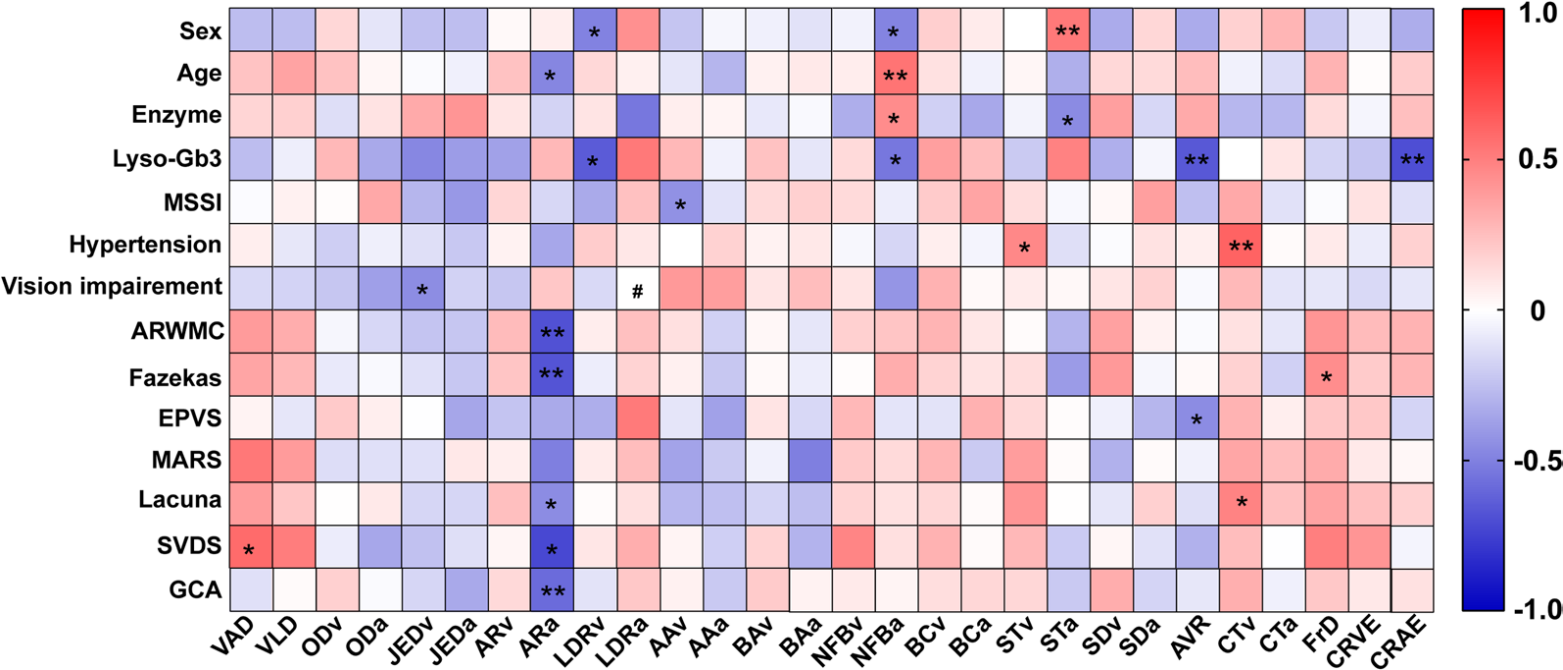
The correlation heat map for RMPs and systemic parameters and MRI scores. Vertical coordinate represented system parameters and MRI scores, and horizontal ordinate indicated RMPs. The color depth of the ribbon on the right represented the values of the correlation coefficient r, a positive correlation with red and a negative correlation with blue. Correlations are stronger with darker colors. *p < 0.05, **p < 0.01; # data not available

RMPs moderately correlated with systemic parameters and MRI scores were further carried out and presented in a visual correlation heatmap (Fig. 3). Interestingly, the calculated vascular indices showed significant correlations with plasma (STa: *r* =  − 0.459, *p* = 0.037; NFBa: *r* = 0.450, *p* = 0.036) and Lyso-Gb3 levels (CRAE: *r* =  − 0.696, *p* = 0.004; AVR: *r* =  − 0.657, *p* = 0.008; NFBa: *r* =  − 0.535, p = 0.040; LDRv: *r* =  − 0.643, p = 0.024). Furthermore, an asymmetry index was negatively correlated with the MSSI score, a well-established disease severity score for FD assessment (AAv: *r* =  − 0.431, *p* = 0.045). More importantly, there were significant negative correlations between ARa and CSVD-related scores (ARWMC: *r* =  − 0.683, *p* = 0.001; Fazekas: *r* =  − 0.673, *p* = 0.001; Lacuna: *r* =  − 0.453, *p* = 0.045; SVDS: *r* =  − 0.721, *p* = 0.012; GCA: *r* =  − 0.582, *p* = 0.009). Additionally, RMPs were also positively correlated with Fazekas scores (FrD: *r* = 0.446, *p* = 0.037), Lacuna (CTv: *r* = 0.482, *p* = 0.023), and SVDS scores (VAD: *r* = 0.580, *p* = 0.038). An additional table file shows correlation analysis in more detail (Supplementary Table @@1). To determine the ability of RMPs to monitor disease progression, we classified FD patients into two groups: mild (MSSI score < 20) and moderate-to-severe (MSSI score ≥ 20) [39]. Given that only AAv showed a negative correlation with MSSI, ROC curve analysis of AAv was performed. The areas under the curve for the AAv was 0.783 (95% CI, 0.563–1.000; *p* = 0.025), with a specificity of 83.3% and a sensitivity of 80%.

## Discussion

With DL-aided model, we used quantified parameters to objectively describe the morphology and extension of retinal vascular networks. Initially, our findings indicated notable disparities in RMPs between the FD patients and the controls. These disparities encompassed vascular diameter, density, symmetry, bifurcation, and tortuosity. Second, we validated the close relationships of RMPs with brain MRI-related indices, disease markers and severity scores, which will be of great significance for monitoring CSVD-related brain lesions, disease severity and prognosis.

Our research showed that retinal vessels were reduced in size and density in Fabry disease compared to controls. This was due to lower vascular morphological parameters (VLD, VAD, CRAE, and CRVE). The degree of arterial and venous diameter abnormalities was comparable with no disparity in AVR. A decrease in vessel diameter would lead to an increased wall shear stress according to the formula of Poiseuille’s law [40]. Reduced retinal branch network density and diameter can hinder retinal microcirculation and increase damage to the vascular endothelium due to increased shear stress. A study by Minnella AM et al. found that decreased retinal vascular density was linked to reduced focal electroretinography amplitudes, indicating a connection between microangiopathy signs and retinal dysfunction [8].

Our results further revealed that venular tortuosity parameters were significantly higher in FD patients compared to the control group, increasing by about 1.5 times. This was due to the buildup of Gb3 in vascular endothelial cells, leading to decreased vascular wall resistance to hydrostatic pressure [6–8]. In α-Gal A-knockout mice, Gb3 accumulation not only reduced K(Ca)3.1 channel activity but also accelerated the breakdown of endothelial K(Ca)3.1 in lysosomes [5, 41]. Severe endothelial dysfunction and vasculopathy occurred in FD [5, 41]. Arterial tortuosity increased, but not significantly. Limited instances made it difficult to distinguish between normal and irregular arterioles. Arteries have fast blood flow, elasticity, and strong resistance to pressure, which may explain why Gb3 has less impact on arteries than veins [42]. Furthermore, within the FD group, males exhibited higher vascular tortuosity than females, match the previous report [43]. The more severe systemic and ophthalmologic phenotype is seen in males, which may be attributed to the X-linked inheritance pattern [1, 15, 16].

In FD patients, there was a decrease in arteriolar BA and venular AA, as well as an increase in arteriovenous AR. These alterations in vascular extension may be linked to vessel damage from GB3 aggregation. Reduced FrD suggests easier retinal vascular stratification in FD compared to healthy individuals. JED and OD were ineffective in distinguishing between FD patients and controls or between male and female FD patients. Both α-Gal A and Lyso-Gb3 levels were important for diagnosing and assessing the severity of FD. The close relationship between Lyso-Gb3 levels and hyperreflective foci supported the theory that vascular changes in FD were due to Gb3 accumulation [11, 43]. The current investigation demonstrated that RMPs (e.g., diameter, the number of first branches and tortuosity) of Fabry disease were closely correlated with Lyso-Gb3 levels and α-Gal A. The assessment found that venular asymmetry was negatively correlated with disease severity, as measured by MSSI scores. This contradicted previous studies, possibly due to differences in sample size and patient characteristics [9, 15]. Nonetheless, these results suggest that vascular parameters can be used to monitor disease progression in FD patients.

Changes in retinal microvessels as early indicators of FD may suggest a disruption in the function of the microvascular system. Given the anatomical and physiological similarities between retinal and cerebral vessels [21, 22], the RMPs could serve as a monitoring window for cerebral microvascular disorders and cortical damage. Therefore, we further explored the correlation between RMPs and CSVD scores based on brain MRI imaging.

Our research results show that RMPs in FD patients, especially the increase in retinal ARa, were negatively correlated with all CSVD-related scores except MARS, including ARWMC, Lacuna, EPVS, SVDS, and GCA. It suggested that retinal arteriolar asymmetry is associated with WMHs, LIs, and brain atrophy and may serve as a predictive factor for them. WMHs is typically correlated with focal chronic gray matter ischemia. Our study found that which WMHs was associated with lower FrD as well, which was consistent with the report by McGrory, S et al. in the Mild Stroke Study [44]. In another cohort study of monozygotic twins, van de Kreeke, J.A. et al. revealed an association between the volume of deep WMHs, increased arterial FrD, and retinal venous tortuosity [45]. These scholars believed that this association may be primarily driven by shared genetic factors and unique environmental factors [45]. LIs refers to small infarcts with size of 2 to 20 mm in deep brain. In addition to retinal arteriolar asymmetry, LIs was also associated with increased venous tortuosity. In vascular density parameters, we revealed decreased VAD was related to brain atrophy in our research, while Cennamo, G. et al. showed that FD patients with neurological issues had a lower vascular density in the superficial capillary plexus compared to those without neurological issues [4]. Indeed, CSVD-related lesions indicated by clinical MRI directly reflect irreversible brain damage patterns rather than the composition and lesions of small vessels within the brain.

The results of the ROC curve analysis indicated that AAv has moderate performance in identifying mild and moderate-to-severe FD patients and could serve as a reliable biomarker for monitoring disease progression in FD. Furthermore, the RMPs markers were of particular interest because their measurement and data collection are non-invasive. While MRI examinations are costly, complex to analyze, and time-consuming, fundus photography has the advantages of being inexpensive, fast, and non-invasive. Therefore, fundus photography combined with AI-assisted analysis holds significant clinical importance and potential value in monitoring microvascular alterations and disease progression in clinical practice, providing clinicians with a basis to better manage FD patients. Future multicenter prospective observational studies with larger sample sizes are needed to confirm RMPs as predictive biomarkers.

Admittedly, there are some limitations to our study. First, our findings based on a cross-sectional design require further cohort studies to establish a stronger causal relationship.

In the correlation analysis, the observed correlation of ARa and CSVD-related scores could be confounded by age, as ARa is also negatively correlated with age. Furthermore, the relationships for some of parameters and scores were significant but with very low correlation coefficient. Given these limitations, we plan to conduct a larger sample FD cohort study in the future to acquire more robust relationship. Fundus photography has the advantages of rapid and non-invasive, but it is limited to the analysis of retinal arterioles and venules. In the future, Optical coherence tomography angiography, another common noninvasive imaging technique, can be used to further explore the relationship between capillary system, choroidal changes and brain lesions. The ocular refractive error without adjustment and some included FD patients with hypertension were other potential limitations to the study. Furthermore, it would be much valuable to research the effect of enzyme replacement therapy on vasculopathy. However, none of the FD patients had received to enzyme therapy during the data collection phase of our study. Despite these limitations, our available evidence for the first time supported retinal microvascular network as a potential tool for identifying early brain lesions in FD, providing a basis for further research and clinical practice.

## Conclusion

In conclusion, our quantitative evaluation of retinal vasculature using fundus photographs demonstrated increased vessel tortuosity and asymmetry, decreased density, diameter and branching angle, and simpler vascular stratification in Fabry. Supported by AI-aided image segmentation and quick evaluation, the noninvasive and rapid acquisition of fundus images may help preliminarily assess CSVD-related brain lesions and monitor the disease severity and progression for FD diseases in the future.

## Supplementary Information

Additional file1 (DOCX 27 KB)

## Data Availability

The data that support the findings of this study are not publicly available due to the containing information that could compromise the privacy of research participants but are available from the corresponding author [Yuan Wu, email: wuyuan@pku.edu.cn] upon reasonable request.
