# Supplementary material for: Deep learning assisted retinal microvasculature assessment and cerebral small vessel disease in Fabry disease
Source: Orphanet J Rare Dis. 2025 Apr 3;20:158. doi: 10.1186/s13023-025-03627-1 (PMC11969690; doi:10.1186/s13023-025-03627-1)
Supplement: Supplementary file 1 — Additional file1 (DOCX 27 KB) [file 13023_2025_3627_MOESM1_ESM.docx]

Supplementary Table 1 The correlations of RMPs of FD patients with systemic parameters and MRI scores.

| Parameters | Sex | Age | α-Gal A | Lyso-Gb3 | MSSI | Hypertension | Vision impairment | ARWML | Fazekas | EPVS | MARS | Lacunae | SVDS | GCA |
| --- | --- | --- | --- | --- | --- | --- | --- | --- | --- | --- | --- | --- | --- | --- |
| CRAE | -0.323 | 0.199 | 0.250 | **-0.696^**^** | -0.127 | 0.177 | -0.100 | 0.296 | 0.290 | -0.169 | 0.030 | 0.178 | -0.043 | 0.117 |
| CRVE | -0.073 | 0.012 | -0.040 | -0.229 | 0.112 | -0.080 | -0.150 | 0.266 | 0.200 | 0.214 | 0.084 | 0.245 | 0.413 | 0.090 |
| FrD | -0.219 | 0.294 | 0.138 | -0.175 | -0.019 | 0.080 | -0.100 | 0.414 | **0.446^*^** | 0.216 | 0.327 | 0.361 | 0.499 | 0.215 |
| CTa | 0.289 | -0.138 | -0.285 | 0.099 | -0.118 | 0.018 | -0.107 | -0.099 | -0.185 | 0.065 | 0.253 | 0.240 | -0.007 | -0.061 |
| CTv | 0.177 | -0.057 | -0.283 | 0.000 | 0.336 | **0.611^**^** | 0.274 | 0.117 | 0.173 | 0.297 | 0.347 | **0.482^*^** | 0.257 | 0.312 |
| AVR | -0.333 | 0.257 | 0.332 | **-0.657^**^** | -0.251 | 0.064 | -0.025 | -0.021 | 0.022 | **-0.454^*^** | -0.057 | -0.127 | -0.309 | -0.096 |
| SDa | 0.156 | 0.143 | -0.157 | -0.039 | 0.374 | 0.113 | 0.174 | 0.050 | -0.038 | -0.287 | 0.017 | 0.183 | -0.118 | -0.170 |
| SDv | -0.333 | 0.150 | 0.375 | -0.314 | 0.025 | -0.016 | 0.100 | 0.365 | 0.398 | -0.059 | -0.307 | -0.100 | 0.032 | 0.319 |
| STa | **0.522^**^** | -0.317 | **-0.459^*^** | 0.490 | -0.031 | -0.129 | 0.027 | -0.297 | -0.395 | 0.010 | 0.014 | 0.002 | -0.206 | -0.215 |
| STv | 0.000 | 0.034 | -0.049 | -0.211 | 0.133 | 0.467^*^ | 0.075 | 0.013 | 0.131 | 0.146 | 0.384 | 0.415 | 0.277 | 0.156 |
| BCa | 0.077 | -0.056 | -0.341 | 0.253 | 0.355 | -0.043 | 0.020 | 0.093 | 0.106 | 0.306 | -0.209 | 0.019 | 0.019 | 0.156 |
| BCv | 0.187 | 0.116 | -0.186 | 0.375 | 0.201 | 0.064 | 0.299 | 0.293 | 0.171 | -0.113 | 0.290 | 0.153 | 0.300 | 0.129 |
| NFBa | **-0.486^*^** | **0.545^**^** | **0.450^*^** | **-0.535^*^** | -0.073 | -0.164 | -0.418 | 0.226 | 0.320 | -0.108 | 0.144 | 0.118 | 0.121 | 0.041 |
| NFBv | -0.054 | 0.069 | -0.319 | 0.142 | 0.142 | -0.034 | 0.105 | 0.181 | 0.011 | 0.278 | 0.206 | 0.166 | 0.475 | 0.081 |
| BAa | -0.115 | 0.084 | -0.027 | -0.099 | 0.183 | 0.087 | 0.259 | -0.100 | -0.073 | -0.155 | -0.505 | -0.255 | -0.299 | 0.050 |
| BAv | -0.062 | 0.053 | -0.093 | 0.236 | 0.142 | 0.048 | 0.100 | 0.029 | 0.021 | 0.103 | -0.057 | -0.170 | 0.170 | 0.201 |
| AAa | -0.038 | -0.293 | 0.041 | -0.055 | -0.110 | 0.173 | 0.378 | -0.182 | -0.222 | -0.371 | -0.209 | -0.251 | -0.190 | -0.214 |
| LDRa | 0.435 | 0.056 | -0.533 | 0.524 | 0.243 | 0.091 | NA | 0.245 | 0.172 | 0.520 | 0.258 | 0.120 | 0.316 | 0.213 |
| LDRv | **-0.492^*^** | 0.152 | 0.103 | -0.643 | -0.334 | 0.198 | -0.149 | 0.068 | -0.074 | -0.321 | 0.078 | 0.015 | 0.098 | -0.113 |
| ARa | 0.064 | **-0.477^*^** | -0.176 | 0.275 | -0.160 | -0.347 | 0.219 | **-0.683^**^** | **-0.673^**^** | -0.335 | -0.505 | **-0.453^*^** | **-0.721^*^** | **-0.582^**^** |
| ARv | 0.021 | 0.237 | 0.101 | -0.371 | 0.159 | 0.048 | -0.224 | 0.265 | 0.224 | -0.234 | 0.064 | 0.250 | 0.038 | 0.148 |
| JEDa | -0.255 | -0.060 | 0.414 | -0.390 | -0.408 | -0.217 | -0.179 | -0.224 | -0.221 | -0.350 | 0.087 | -0.163 | -0.128 | -0.339 |
| JEDv | -0.250 | -0.021 | 0.330 | -0.471 | -0.288 | -0.129 | -0.449^*^ | -0.233 | -0.125 | -0.006 | -0.125 | -0.161 | -0.245 | -0.163 |
| ODa | -0.102 | 0.032 | 0.110 | -0.341 | 0.339 | -0.065 | -0.378 | -0.158 | -0.027 | 0.063 | -0.122 | 0.084 | -0.346 | -0.022 |
| ODv | 0.156 | 0.236 | -0.130 | 0.275 | 0.010 | -0.193 | -0.224 | -0.039 | -0.087 | 0.205 | -0.135 | 0.001 | -0.075 | 0.181 |
| VLD | -0.260 | 0.361 | 0.181 | -0.068 | 0.051 | -0.097 | -0.174 | 0.321 | 0.276 | -0.101 | 0.391 | 0.223 | 0.508 | 0.016 |
| VAD | -0.260 | 0.235 | 0.163 | -0.261 | -0.016 | 0.064 | -0.150 | 0.390 | 0.350 | 0.047 | 0.529 | 0.383 | 0.580^*^ | 0.087 |

RMPs: retinal microvascular parameters; FD: Fabry disease; NA: data not available; MRI: magnetic resonance imaging; α-Gal A: α-galactosidase A; Lyso-Gb3: globotriaosylsphingosine; MSSI: Mainz severity score index; ARWMC: age related white matter changes; EPVS: enlarged perivascular spaces; MARS: microbleed anatomical rating scale; SVDS: small vessel diseases; GCA: global cortical atrophy; SD, standard deviation; CRAE, central retinal artery equivalent; CRVE, central retinal vein equivalent; VLD, vessel length density; VAD, vessel area density; AVR, arteriovenous ratio; LDR: length-diameter ratio; AA, angle asymmetry; AR, asymmetry ratio; OD: optimality deviation; BA, branching angle; BC, branching coefficient; NFB, number of first branches; JED, junctional exponent deviation; CT, curvature tortuosity; ST, simple tortuosity; FD, fractal dimension. The parameter names ending in lowercase 'a' or 'v' correspond to the arteriolar network or venular network, respectively; *p< 0.05, **p< 0.01.
